# Design of a Piezoelectric Multilayered Structure for Ultrasound Sensors Using the Equivalent Circuit Method

**DOI:** 10.3390/s18124491

**Published:** 2018-12-18

**Authors:** Muhammad Shakeel Afzal, Hayeong Shim, Yongrae Roh

**Affiliations:** School of Mechanical Engineering, Kyungpook National University, Daegu 41566, Korea; shakil.afzal92@yahoo.com (M.S.A.); hiyo3@naver.com (H.S.)

**Keywords:** ultrasonic sensor, equivalent circuit, multilayered structure, acoustical characteristics

## Abstract

This study investigates the electroacoustic behavior of a piezoelectric multilayered structure for ultrasonic sensors using the equivalent circuit method (ECM). We first derived the vertical deflection of the multilayered structure as a function of pressure and voltage using equilibrium equations of the structure. The deflection derived in this work is novel in that it includes the effect of piezoelectricity as well as the external pressure on the radiating surface. Subsequently, the circuit parameters were derived from the vertical deflection. The acoustic characteristics of the structure were then analyzed using the electro-acoustical model of an ultrasonic sensor for in-air application. Using the equivalent circuit, we analyzed the effects of various structural parameters on the acoustic properties of the structure such as resonance frequency, radiated sound pressure, and beam pattern. The validity of the ECM was verified initially by comparing the results with those from the finite element analysis (FEA) of the same structure. Furthermore, experimental testing of an actual ultrasonic sensor was carried out to verify the efficacy of the ECM. The ECM presented in this study can estimate the performance characteristics of a piezoelectric multilayered structure with high rapidity and efficiency.

## 1. Introduction

Ultrasonic sensors in air have a variety of applications like distance measurement for autonomous vehicles, robotics, and consumer electronics due to such features as compact size, high reliability, and low power consumption [1,2,3,4,5,6]. Most in-air ultrasonic sensors use piezoceramics as their transduction material and have typically a multilayered structure: a piezoelectric disc which vibrates radially, a metallic plate that vibrates in bending mode due to clamped edges, a bonding layer to join the piezoceramic disc with the metallic plate, and a backing layer to absorb the ultrasound waves propagating backward [7,8,9,10]. The performance of the ultrasonic sensor is highly dependent on dimensions, boundary conditions, and the material properties of each layer. In order to develop a good ultrasonic sensor, the performance characteristics of the multilayered structure should be analyzed meticulously.

Research on various structural configurations of piezoelectric ultrasonic sensors has extensively been performed both analytically and experimentally. Stavsky and Loewy derived the equations of motion and presented dynamic responses of non-piezoelectric composite circular plates [11]. Morris and Forster used the finite element method (FEM) to optimize the deflection of a circular piezoelectric plate in terms of the actuator plate stiffness, radius ratio, and the bonding layer thickness for fixed and pinned edge conditions [12]. A variety of coupled domain models of a piezoelectric transducer with different shapes have been proposed over the past several years [13,14,15,16]. Prasad et al. proposed an electro-acoustic model for a unimorph structure comprising a piezoelectric circular plate bonded to a magnesium plate [17]. They studied the deflection of a simplified two-layered structure in terms of its thickness and radius ratio. However, their study was limited to the analysis of vertical deflection only and did not analyze the acoustic characteristics of the structure such as electromechanical impedance and radiated sound pressure. Araromi and Burgess analyzed a unimorph dielectric elastomer actuator with inhomogeneous layer geometry using the FEM [18]. Liu et al. dealt with the behavior of a piezoelectric unimorph circular plate and examined the vibrational response of a clamped plate [19]. Li and Chen also presented analytical calculations regarding the performance of a partially covered piezoelectric circular actuator [20]. They discussed some parametric studies to optimize the actuator design. The effect of edge conditions for circular diaphragm actuators with full and partial piezoelectric coverage was examined by Mo et al. [21]. However, these works considered a particular, simplified multilayered structure like the unimorph, not a generalized structure composed of an arbitrary number of multiple layers.

As illustrated in the above discussion, the acoustical characteristics of the piezoelectric multilayered structure could be analyzed using different methods. These methods include a theoretical analysis method, equivalent circuit method (ECM) and FEM [22,23,24]. The theoretical analysis for in-air ultrasound radiation was carried out in our previous study as well [25]. However, the pure theoretical method had a limitation in its accuracy because of the inevitable simplification of physical parameters, which motivated the development of a more accurate and reliable method to predict the performance of the structure.

The ECM is a promising technique to analyze the piezoelectric transducers comprised of various shapes and arrangements of the piezoceramic and the adjacent backing and matching layers [26,27]. The Mason’s equivalent circuit, the Krimholtz-Leedom-Matthae (KLM) model and their extended versions have been adopted in various studies to analyze the electromechanical behavior of the acoustical transducers having multiple layers [28,29,30].

In this work, we develop an electroacoustic equivalent circuit for a more efficient and reliable estimation of the characteristics of the piezoelectric multilayered structure as an in-air ultrasonic sensor in the frequency range of several and several tens of kHz. The ECM includes the effect of piezoelectricity as well as the external pressure on the radiating surface. Moreover, performance characteristics such as the input impedance of the ultrasonic sensor can also be directly calculated using the ECM. Circuit parameters are extracted from the vertical displacement, which is derived from equilibrium equations, of the structure. Using the equivalent circuit, we analyze the effects of various structural parameters on the acoustic properties of the structure such as resonance frequency and electromechanical impedance. Acoustical beam pattern of the structure is also derived on the basis of the equivalent circuit analysis (ECA) results. Results of the ECA is validated first numerically using the FEM to compare resonance frequency and radiated pressure variations with reference to dimensional variations. Then an experiment is carried out to ensure a more realistic validation of the ECM by comparing the impedance spectrum and beam pattern of an actual multilayered ultrasonic sensor with those from the ECA. The ECM presented in this work can estimate the sensor performance accurately with high rapidity and efficiency than existing methods like the FEM and the precedent theoretical method of the authors [25].

## 2. Analysis of the Piezoelectric Multilayered Structure

**Figure 1:**
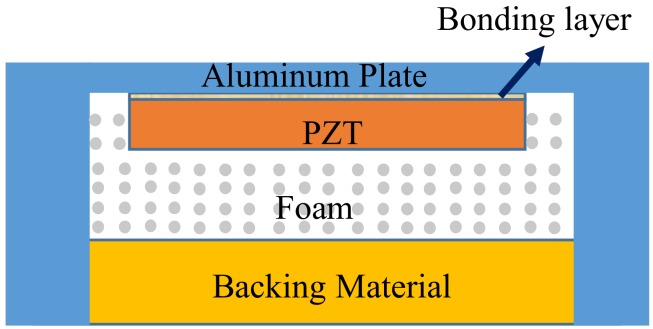
The cross sectional view of a typical ultrasonic sensor.

**Figure 2:**
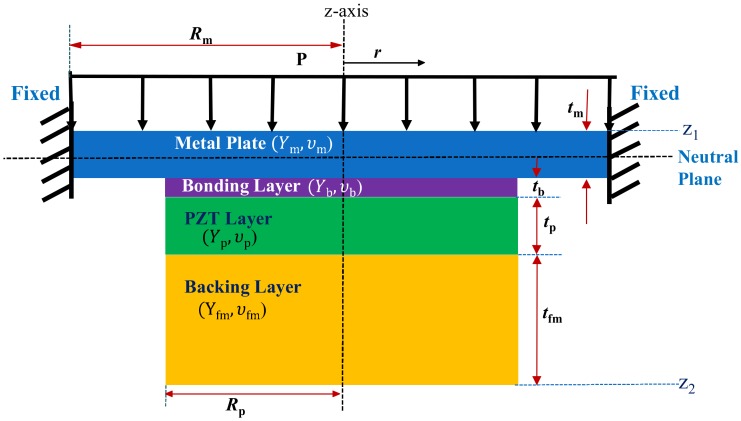
Schematic of the axisymmetric multilayered structure in the z-r plane.

Figure 1 illustrates the cross-sectional view of a typical ultrasonic sensor for in-air applications, which can be simplified as the multilayered composite structure in Figure 2.

The radii of the piezoelectric disc, metallic plate and bonding layer are much larger than their thicknesses; therefore, they can be treated as thin plates with axis symmetry. Surrounding edges of the metallic plate are fixed due to the stiff enclosing case. The z-axis is perpendicular to the plane of vibration whereas *r* and *θ* correspond to the radial and circumferential coordinates of the multilayered structure, respectively. The multilayered structure is composed of two regions, that is, the inner region or region-1 containing the multiple layers (0 ≤ *r* ≤ *R_p_*) and the annular region or region-2 containing only the metallic plate (*R_p_* ≤ *r* ≤ *R_m_*). Figure 2 is the schematic model of the multilayered structure in the z-r plane with corresponding material constants.

Composition of the equivalent circuit to analyze the multilayered structure requires determination of the vertical deflection of the structure as a function of pressure and voltage loading. Figure 2 actually corresponds to the cross-section of a clamped circular composite piezoelectric plate which is subjected to a uniform vertical pressure *P* and an electric voltage *V*. Classical laminated plate theory was adopted to derive the equations of equilibrium for the circular composite plate [31,32,33]. The equilibrium equations for a typical axisymmetric plate structure are Equations (1)–(3).

(1) $$\frac{dN_{r}}{dr}+\frac{1}{r}\left(N_{r}-N_{\theta }\right)=0$$

(2) $$\frac{dM_{r}}{dr}+\frac{1}{r}\left(M_{r}-M_{\theta }\right)=F_{r}$$

(3) $$\frac{dF_{r}}{dr}+\frac{F_{r}}{r}+P=0$$

where *N_r_* and *N_θ_* represent the force in radial and circumferential directions, respectively, *M_r_* and *M_θ_* represent the moment in radial and circumferential directions, respectively, and *F*_r_ is a shear force. The radial and circumferential strain-displacement relationships can be described using Kirchoff’s plate theory as Equations (4)–(9) [31].

(4) $$\epsilon_{rr}=\varepsilon_{rr}^{\prime }+zk_{r}$$

(5) $$\epsilon_{\theta \theta }=\varepsilon_{\theta \theta }^{\prime }+zk_{\theta }$$

(6) $$\epsilon_{rr}^{\prime }=\frac{du_{0}}{dr}$$

(7) $$k_{r}=-\frac{d\theta }{dr}$$

(8) $$\epsilon_{\theta \theta }^{\prime }=\frac{u_{0}}{r}$$

(9) $$k_{\theta }=-\frac{\theta }{r}$$

where *ε_rr_* and *ε_θθ_* are radial and circumferential strains at a point of interest, respectively, and *k_r_* and *k_θ_* are radial and circumference curvatures, respectively. *ε′_rr_* and *ε′_θθ_* are radial and circumferential strains at the neutral plane (z = 0), respectively, while *u* and *θ* represent the radial displacement and vertical deflection, respectively.

The general piezoelectric relationship between strain *ε* and stress *σ* is given by Equation (10) [34].

(10) $$R\left[\sigma \right]=[c^{E}\left[\epsilon \right]-\left[e\right]\left[E\right]$$,

where *E* represents electric field, *c^E^* elastic stiffness measured at constant *E*, and *e* piezoelectric stress constant. The general piezoelectric constitutive equation can be extended to the axisymmetric multilayered structure in Figure 2 as Equation (11) [17].

(11) $$\left\{\begin{array}{c} \sigma_{rr}\\ \sigma_{\theta \theta } \end{array}\right\}=\left[Q_{x}\right]\left(\left\{\begin{array}{c} \varepsilon_{rr}^{\prime }\\ \varepsilon_{\theta \theta }^{\prime } \end{array}\right\}+z\left\{\begin{array}{c} k_{r}\\ k_{\theta } \end{array}\right\}-E_{3}\left\{\begin{array}{c} d_{31}\\ d_{31} \end{array}\right\}\right)$$

where *σ_rr_* and *σ_θθ_* represent the radial and the circumferential stresses, respectively, *E*_3_ is the electric field applied along the *z*-axis, and *d*_31_ is the piezoelectric constant connecting the *E*_3_ with the transverse stresses. The [*d*] constant is related to the [*e*] constant as [*e*] = [*d*][*c^E^*] [35]. For the layers other than the piezoelectric layer, the piezoelectric constant *e* is absent, which simplifies Equation (11) to the form that does not have the electric field. The term *Q_x_* corresponds to the reduced stiffness coefficient for each layer of the composite structure and can be defined as Equation (12).

(12) $$\left[Q_{x}\right]=\frac{Y_{x}}{1-\nu_{x}^{2}}\left\{\begin{array}{cc} 1 & \nu_{x}\\ \nu_{x} & 1 \end{array}\right\}$$

where *Y_x_* is the Young’s modulus and *υ_x_* is the Poisson’s ratio of respective layer. Using Equation (12), the reduced stiffness coefficients of the metallic circular plate (*Q_m_*), bonding layer (*Q_b_*), piezoelectric disk (*Q_p_*) and backing layer (*Q_fm_*) are calculated. Further, the radial and circumferential forces and moments in Equations (1)–(3) can be obtained by integrating the constitutive Equation (11) as given by Equations (13) and (14) [17].

(13) $$\left\{\begin{array}{c} N_{r}\\ N_{\theta } \end{array}\right\}=\left(\left[A\right]\left\{\begin{array}{c} \varepsilon_{rr}^{\prime }\\ \varepsilon_{\theta \theta }^{\prime } \end{array}\right\}+\left[B\right]\left\{\begin{array}{c} k_{r}\\ k_{\theta } \end{array}\right\}-\left\{\begin{array}{c} N_{r}^{p}\\ N_{\theta }^{p} \end{array}\right\}\right),$$

(14) $$\left\{\begin{array}{c} M_{r}\\ M_{\theta } \end{array}\right\}=\left(\left[B\right]\left\{\begin{array}{c} \varepsilon_{rr}^{\prime }\\ \varepsilon_{\theta \theta }^{\prime } \end{array}\right\}+\left[D\right]\left\{\begin{array}{c} k_{r}\\ k_{\theta } \end{array}\right\}-\left\{\begin{array}{c} M_{r}^{p}\\ M_{\theta }^{p} \end{array}\right\}\right)$$

where [*A*], [*B*], and [*D*] are extensional stiffness, coupling flexural-extensional stiffness, and flexural stiffness terms that can be combined for multilayered structure and presented as Equations (15)–(17).

(15) $$\begin{array}{ll} \left[A\right]=\overset{Z_{2}}{\underset{Z_{1}}{\int }}\left[Q_{x}\right]d_{z} & =\left[Q_{m}\right]\times \left(Z_{i}-Z_{1}\right)+\left[Q_{b}\right]\times \left(Z_{i1}-Z_{i}\right)+\left[Q_{p}\right]\times \left(Z_{i2}-Z_{i1}\right)\\ & +\left[Q_{fm}\right]\times \left(Z_{2}-Z_{i2}\right) \end{array}$$

(16) $$\begin{array}{ll} \left[B\right]=\overset{Z_{2}}{\underset{Z_{1}}{\int }}\left[Q_{x}\right]zd_{z} & =\left[Q_{m}\right]\times \left(\frac{Z_{i}^{2}-Z_{1}^{2}}{2}\right)+\left[Q_{b}\right]\times \left(\frac{Z_{i1}^{2}-Z_{i}^{2}}{2}\right)+\left[Q_{p}\right]\times \left(\frac{Z_{i2}^{2}-Z_{i1}^{2}}{2}\right)\\ & +\left[Q_{fm}\right]\times \left(\frac{Z_{2}^{2}-Z_{i2}^{2}}{2}\right) \end{array}$$

(17) $$\begin{array}{ll} \left[D\right]=\overset{Z_{2}}{\underset{Z_{1}}{\int }}\left[Q_{x}\right]z^{2}d_{z} & =\left[Q_{m}\right]\times \left(\frac{Z_{i}^{3}-Z_{1}^{3}}{3}\right)+\left[Q_{b}\right]\times \left(\frac{Z_{i1}^{3}-Z_{i}^{3}}{3}\right)+\left[Q_{p}\right]\times \left(\frac{Z_{i2}^{3}-Z_{i1}^{3}}{3}\right)\\ & +\left[Q_{fm}\right]\times \left(\frac{Z_{2}^{3}-Z_{i2}^{3}}{3}\right) \end{array}$$

The terms *N_r_^P^*, *N_θ_^P^, M_r_^P^*, and *M_θ_^P^* in Equations (13) and (14) represent the forces and moments generated due to the piezoelectric effect. Substitution of *N_r_^P^*, *N_θ_^P^, M_r_^P^*, and *M_θ_^P^* into Equations (1)–(3) and simplification yields the governing equations for the multilayered structure expressed as Equations (18) and (19) [17,21].

(18) $$\frac{d^{2}u\left(r\right)}{dr^{2}}+\frac{1}{r}\frac{du\left(r\right)}{dr}-\frac{u\left(r\right)}{r^{2}}=\frac{Pr\left(\frac{B_{11}}{A_{11}}\right)}{2\left(D_{11}-\left(\frac{B_{11}^{2}}{A_{11}}\right)\right)},$$

(19) $$\frac{d^{2}\theta \left(r\right)}{dr^{2}}+\frac{1}{r}\frac{d\theta \left(r\right)}{dr}-\frac{\theta \left(r\right)}{r^{2}}=\frac{Pr}{2\left(D_{11}-\left(\frac{B_{11}^{2}}{A_{11}}\right)\right)}.$$

General solutions of the governing equations are derived as Equations (20) and (21).

(20) $$u\left(r\right)=a_{1}r+\frac{a_{2}}{r}-\frac{Pr^{3}\left(\frac{B_{11}}{A_{11}}\right)}{16\left(D_{11}-\left(\frac{B_{11}^{2}}{A_{11}}\right)\right)},$$

(21) $$\theta \left(r\right)=b_{1}r+\frac{b_{2}}{r}-\frac{Pr^{3}}{16\left(D_{11}-\left(\frac{B_{11}^{2}}{A_{11}}\right)\right)}$$

where *a*_1_, *a*_2_, *b*_1_, and *b*_2_ are constants to be evaluated for inner and outer regions of the composite structure. *A*_11_, *B*_11_ and *D*_11_ are constant terms corresponding to the extensional, flexural-extensional coupling, and flexural stiffness matrices of the composite structure. The solution of the governing equations for the inner and outer regions requires the determination of eight constants using boundary conditions and interface matching conditions for the multilayered structure shown in Figure 2. The boundary conditions at the center of the structure and the fixed ends are expressed as Equations (22)–(25).

(22) θ(0)=finite,

(23) u(0)=finite,

(24) $$u{|}_{r=R_{m}}=0,$$

(25) $$\theta \theta {|}_{r=R_{m}}=0.$$

Similarly, the boundary conditions at the interface of the inner and outer regions are expressed as Equations (26)–(29).

(26) $$\theta_{1}{|}_{r=R_{p}}=\theta_{2}{|}_{r=R_{p}},$$

(27) $$u_{1}{|}_{r=R_{p}}=u_{2}{|}_{r=R_{p}},$$

(28) $$N_{r\left(1\right)}{|}_{r=R_{p}}=N_{r\left(2\right)}{|}_{r=R_{p}},$$

(29) $$M_{r\left(1\right)}{|}_{r=R_{p}}=M_{r\left(2\right)}{|}_{r=R_{p}}$$

where subscripts (1) and (2) correspond to the inner composite region and outer metallic annular region, respectively. The vertical deflection of the multilayered structure *W(r)* is calculated by integrating the slope *θ(r)* with respect to the radius. The final functional forms of the vertical deflection at the inner composite region and the outer homogeneous region are derived as Equations (30) and (31), respectively.

(30) $$\begin{array}{c} W_{1}\left(r\right)=b_{1}^{\left(1\right)}\left(\frac{r^{2}-R_{p}^{2}}{2}\right)-\frac{P\left(r^{2}-R_{p}^{2}\right)}{64\left(D_{11}^{\left(1\right)}-\left(\frac{B_{11}^{2\left(1\right)}}{A_{11}^{\left(1\right)}}\right)\right)}+b_{1}^{\left(2\right)}\left[\frac{R_{p}^{2}-R_{m}^{2}}{2}-R_{m}^{2}\ln\left(\frac{R_{p}}{R_{m}}\right)\right]\\ +\frac{P}{64D_{11}^{\left(2\right)}}\left[4R_{m}^{4}\ln\left(\frac{R_{p}}{R_{m}}\right)-R_{p}^{4}+R_{m}^{4}\right], \end{array}$$

(31) $$W_{2}\left(r\right)=b_{1}^{\left(2\right)}\left[\frac{r^{2}-R_{m}^{2}}{2}-R_{m}^{2}\ln\left(\frac{r}{R_{m}}\right)\right]+\frac{P}{64D_{11}^{\left(2\right)}}\left[4R_{m}^{4}\ln\left(\frac{r}{R_{m}}\right)-r^{4}+R_{m}^{4}\right]$$

where *b_1_^(1)^*, and *b_1_^(2)^* are constants corresponding to the inner and outer regions of the composite structure, respectively. The total vertical deflection of the multilayered structure can be expressed as a combination of the deflections of the two regions as given by Equation (32).

(32) $$W\left(r\right)=W_{1}\left(r\right)|\begin{array}{c} R_{p}\\ 0 \end{array}+W_{2}\left(r\right)|\begin{array}{c} R_{m}\\ R_{p} \end{array}.$$

## 3. ECA of the Multilayered Structure

**Figure 3:**
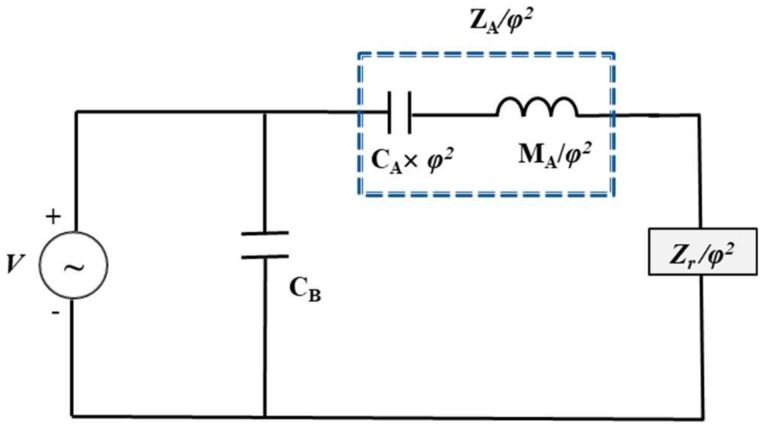
Equivalent circuit of the multilayered structure.

The equivalent circuit for the multilayered structure in Figure 2 is shown in Figure 3, where *C_A_* is acoustic compliance, *M_A_* is acoustic mass, *C_B_* is mechanically blocked electrical capacitance, *Z_r_* is radiation impedance, *φ* is a turning ratio, and *Z_A_* is acoustic impedance. The electrical and acoustical damping terms are neglected for simplicity.

The input admittance *Y_IN_* of the multilayered structure is given by Equation (33) [34].

(33) $$Y_{IN}=i\omega C_{B}+\frac{{\left(-\frac{d_{A}}{C_{A}}\right)}^{2}}{\left(i\left(\omega M_{A}-\frac{1}{\omega C_{A}}\right)\right)+\left(R_{r}+iX_{r}\right)}$$

where *ω* is angular frequency. *φ* is presented as *−d_A_/C_A_* where *d_A_* is an effective piezoelectric constant and *Z_r_* is presented as *R_r_* + *iX_r_* where *R_r_* is radiation resistance and *X_r_* is radiation reactance [36].

The piezoelectric composite structure can be lumped as equivalent circuit elements using electroacoustic analogy. In this electroacoustic analogy, differential pressure and volumetric flow rate are analogous to voltage and current, respectively. The one-dimensional time harmonic piezoelectric coupling equation for our model is Equation (34) [37].

(34) $$\left\{\begin{array}{c} U\\ I \end{array}\right\}=\left[\begin{array}{cc} i\omega C_{A} & i\omega d_{A}\\ i\omega d_{A} & i\omega C_{0} \end{array}\right]\left\{\begin{array}{c} P\\ V \end{array}\right\}$$

(35) $$U=i\omega \Delta Vol=i\omega C_{A}P+i\omega d_{A}V$$

where *U* is volume velocity, *ΔVol* is volume displacement, *I* is current, *C*_0_ is mechanically free electrical capacitance that is related to the blocked electrical capacitance as *C_B_* = *C_0_*(1 − *k*^2^), and *k* is the electromechanical coupling factor of the piezoelectric layer. The volume displacement caused by the vibrational plate is given by

(36) $$\Delta Vol=\overset{2\pi }{\underset{0}{\int }}\overset{r}{\underset{0}{\int }}W\left(r\right)rdrd\theta =2\pi \overset{r}{\underset{0}{\int }}W\left(r\right)rdr\Delta$$

The parameters *C_A_* and *d_A_* can be calculated by applying a unit pressure and a unit voltage individually. The short-circuit acoustic compliance is determined by integrating the vertical deflection generated by the unit pressure and the final functional form of the acoustic compliance is derived as Equation (37).

(37) $$C_{A}=\frac{2\pi }{P}\left(b_{1}^{\left(1\right)}\left(\frac{-R_{p}^{4}}{8}\;\right)+b_{1}^{\left(2\right)}\left(\frac{R_{m}^{2}-R_{p}^{2}}{8}\right)+\frac{PR_{p}^{6}}{192\left(D_{11}^{\left(1\right)}-\left(\frac{B_{11}^{2\left(1\right)}}{A_{11}^{\left(1\right)}}\right)\right)}\;+\frac{P\left(-R_{p}^{4}+\;3R_{m}^{4}-2R_{m}^{6}/R_{p}^{2}\right)}{192\left(D_{11}^{\left(1\right)}-\left(\frac{B_{11}^{2\left(1\right)}}{A_{11}^{\left(1\right)}}\right)\right)}\right)$$

The effective piezoelectric constant *d_A_* is obtained from the volume displacement due to the unit voltage and the final functional form of *d_A_* is derived as Equation (38).

(38) $$d_{A}=\frac{2\pi }{V}\left(b_{1}^{\left(1\right)}\left(\frac{-R_{p}^{4}}{8}\;\right)+b_{1}^{\left(2\right)}\left(\frac{-R_{p}^{2}}{2}\;\right)\left(\frac{R_{p}^{2}-R_{m}^{2}}{2}\;-R_{m}^{2\;}\ln\left(\frac{R_{p}}{R_{m}}\right)\right)\;+b_{1}^{\left(2\right)}\left(\frac{R_{m}^{4}-R_{p}^{4}}{8}+\frac{R_{m}^{2}R_{p}^{2}}{2}\ln\left(\frac{R_{p}}{R_{m}}\right)\right)\right)$$

The effective acoustic mass (*M_A_*) is then obtained by equating the kinetic energy of the distributed system to that of the lumped acoustic mass and can be expressed as Equation (39) [37].

(39) $$M_{A}=\frac{2\pi }{C_{A}^{2}}\int_{0}^{R_{m}}\rho_{A}{\left(\frac{W_{1}\left(r\right)+W_{2}\left(r\right)}{P}{|}_{V=0}\right)}^{2}rdr$$

where *ρ_A_* is the density of the corresponding layer.

Once these circuit parameters are determined, the resonance frequency *f_r_* of the circuit can be determined as Equation (40).

(40) $$f_{r}=\frac{1}{2\pi }\left(\frac{-X_{r}M_{A}\pm \sqrt{{\left(X_{r}M_{A}\right)}^{2}+4\left(C_{A}M_{A}\right)}}{2(C_{A}M_{A})}\right)$$

## 4. Sound Pressure from the Multilayered Structure

The time harmonic form of the vertical deflection given by Equation (32) can be written as Equation (41).

(41) $$W\left(r,t\right)=W\left(r\right)e^{i\omega t}$$

The particle velocity *v*(*r*,*t*) corresponding to the vertical deflection can be obtained by differentiating the displacement and is expressed as

(42) v(r,t)=iωW(r,t).

The sound pressure radiated into air caused by the vibrational plate can be obtained by the Rayleigh integral of the particle velocity as shown in Equation (43) [36].

(43) $$P_{r}\left(r_{d},t\right)=-i\frac{\rho_{air}\omega_{r}}{2\pi r_{d}}\int_{n}v\left(r,t\right)e^{i\left(-k_{air}r_{d}\right)}dn$$

where *ω*_r_ is the angular resonance frequency that is the product of 2π and Equation (40), ρ_air_ is the density of air and *k*_air_ is the wave number. *r*_d_ is a far-field point in air from the vibrational plate, which is maintained to be 1 m throughout the analyses. The sound pressure radiated to a far-field point is evaluated with Equation (43) in relation to the structural parameters of the piezoelectric multilayered structure.

## 5. Characteristics Analysis of the Multilayered Structure

**Figure 4:**
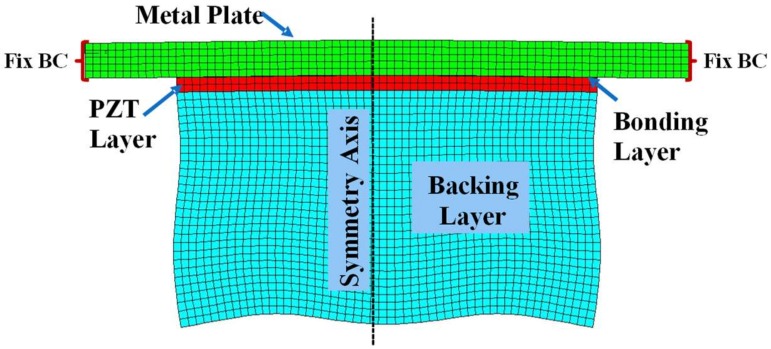
A 2D axisymmetric finite element analysis (FEA) model of the piezoelectric multilayered structure and its deformed shape.

Detailed derivation of the vertical deflection and equivalent circuit parameters was carried out in Section 2 and Section 3, respectively. The effect of structural parameters on the acoustic properties of the piezoelectric multilayered structure was then analyzed with the equivalent circuit in Figure 3. The validity of the analysis is verified by comparing the analysis results with those from the finite element analysis (FEA) of the same structure. The FEA is conducted with the commercial software package PZFlex^®^ (Version 2017, Weidlinger Associate, NY, USA). The 2D axisymmetric finite element model of the piezoelectric multilayered structure is shown in Figure 4. The finite element model consists of 2D quadrilateral elements with four nodes having the size of 0.067 mm along both x- and y-axes. The material properties of the multilayered structure are listed in Table 1 and initial dimensions of the layers are listed in Table 2. The metallic plate is made of aluminum and the piezoelectric material is PZT-5A [35]. The Young’s modulus (*Y_p_*) and Poisson’s ratio (*ν_p_*) of the PZT-5A were derived as *Y_p_* = 1/*s*_11_ and *ν_p_* = −*s*_12_/*s*_11_, respectively, where *s*_11_ and *s*_12_ are the elastic compliance constants of the PZT-5A [25,34,35]. All the dimensions and boundary conditions for the FEA are the same as those for the ECA.

**Figure 5:**
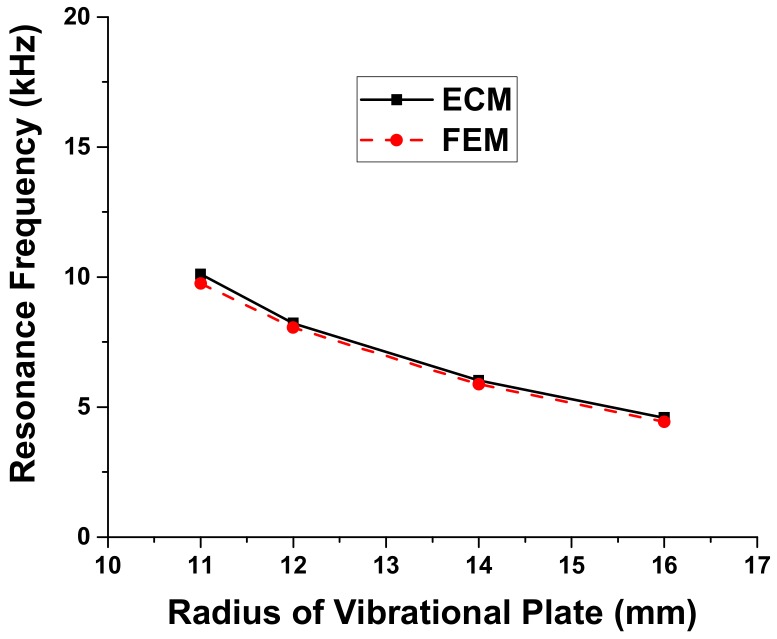
Effect of the vibrational plate radius variation on resonance the frequency.

**Figure 6:**
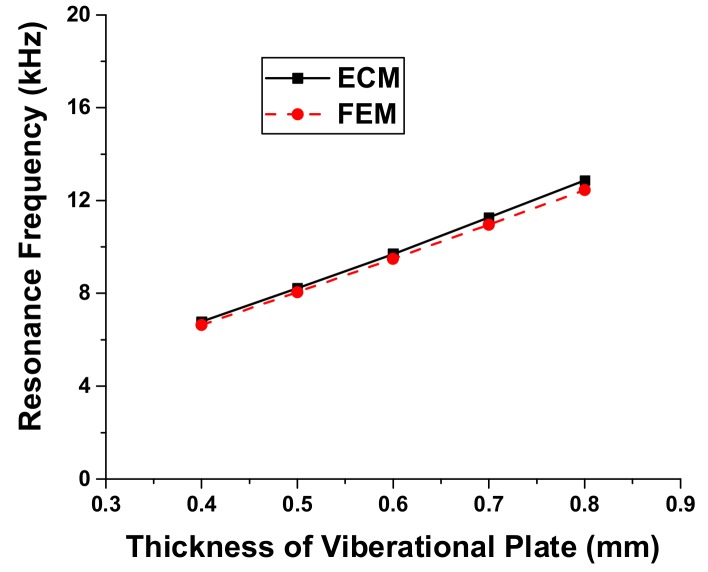
Effect of the vibrational plate thickness variation on the resonance frequency.

Variation of the resonance frequency and radiated sound pressure is first analyzed in relation to the dimension of each layer. Results of the analysis show that the resonance frequency of the multilayered structure is significantly affected by the dimension of the metallic vibrational plate as illustrated in Figure 5 and Figure 6. The effect of dimensional variation of the other three layers on the resonance frequency is almost negligible [25]. Thus, the resonance frequency of the ultrasonic sensor can be effectively controlled by varying the dimension of only the metallic vibrational plate. The comparison between the resonance frequency of the ultrasonic sensor obtained using the ECM and that obtained using the FEM shows excellent agreement as illustrated in Figure 5 and Figure 6. The maximum difference between the two sets of data is 2.1%. The present difference is smaller than the difference obtained using the theoretical method, which was 3.5% [25]. This result confirms that the ECM can provide accurate estimation of the sensor performance with high rapidity and efficiency in comparison with the FEM. Each analysis of the cases in Figure 5 and Figure 6 took less than a minute with the ECM while it took several hours with the FEM.

**Figure 7:**
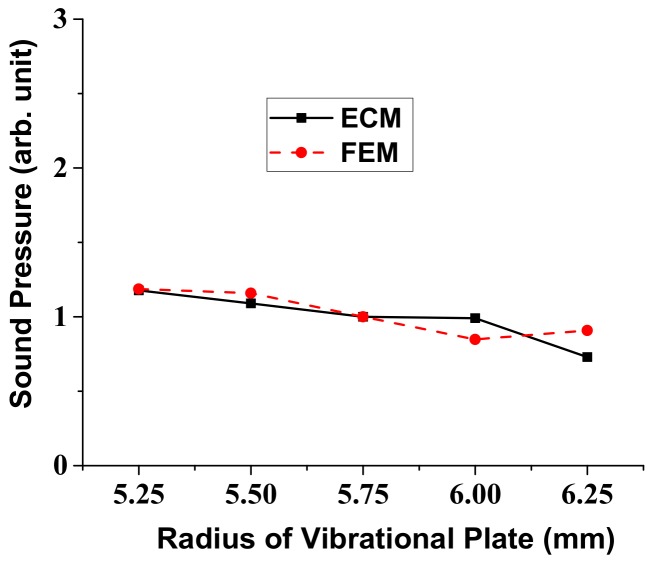
Effect of the vibrational plate radius variation on the radiated sound pressure.

**Figure 8:**
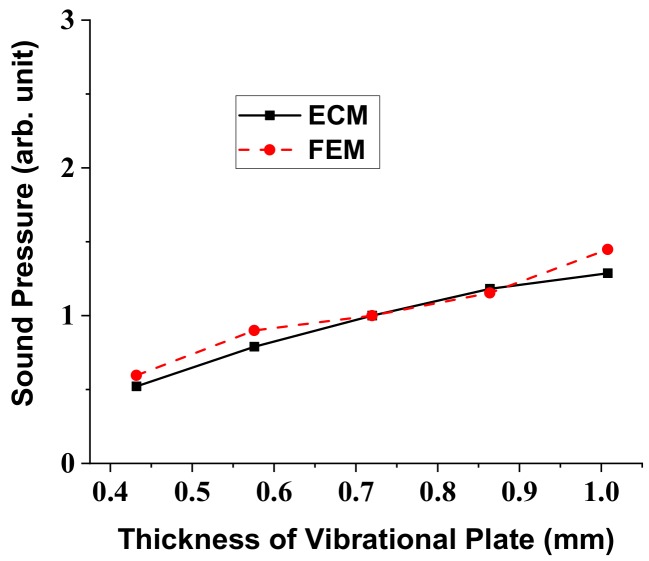
Effect of the vibrational plate thickness variation on the radiated sound pressure.

**Figure 9:**
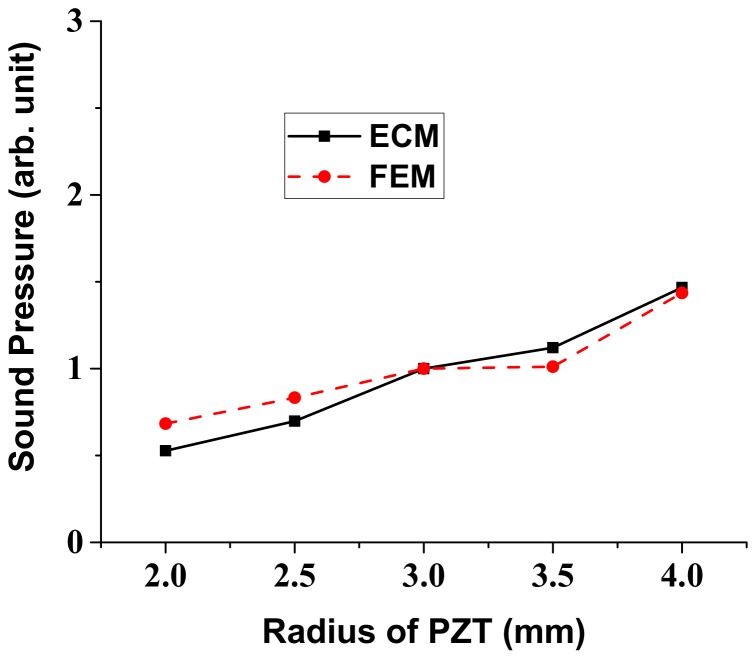
Effect of the PZT radius variation on the radiated sound pressure.

**Figure 10:**
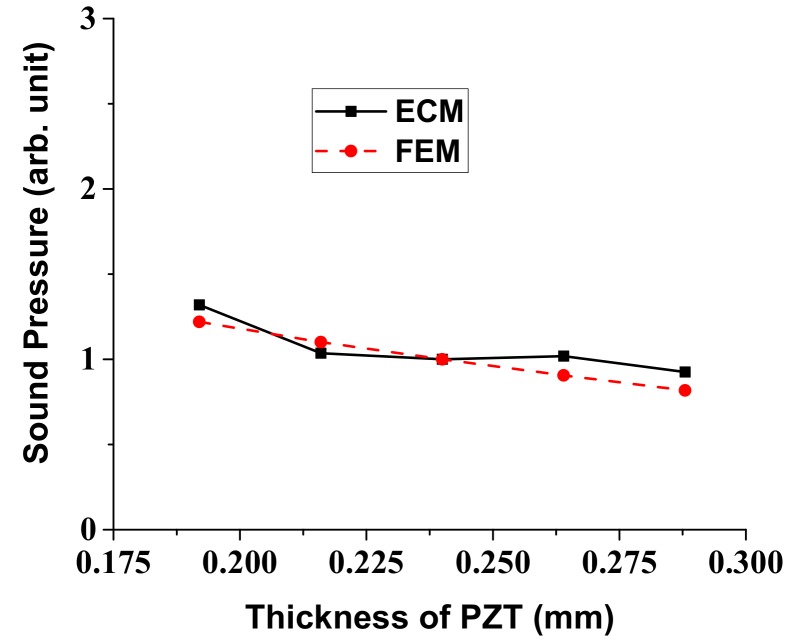
Effect of the PZT thickness variation on the radiated sound pressure.

In a similar way, variation of the radiated sound pressure in relation to the structural parameters of the multilayered structure was analyzed using the ECM. Results of the analysis are shown in Figure 7, Figure 8, Figure 9 and Figure 10, where the sound pressures are normalized to that of the structure having the initial dimension in Table 2. Once we know the response per unit input, the absolute magnitude of the sound pressure can be easily adjusted by just controlling the initial voltage and pressure in the ECA and FEA. The sound pressure turned out to heavily depend on the dimensions of the vibrational plate and the PZT plate but the effect of the other two layers was almost negligible [25]. Here, again, the results obtained by the ECM and the FEM showed good overall agreement with each other. The discrepancy between the two sets of data could be due to the fact that the ECM derivations are based on the thin plate theory that, for simplicity, completely neglects the normal and shear stresses with respect to *z*-axis [32].

## 6. Experimental Measurements of the Actual Ultrasonic Sensor

**Figure 11:**
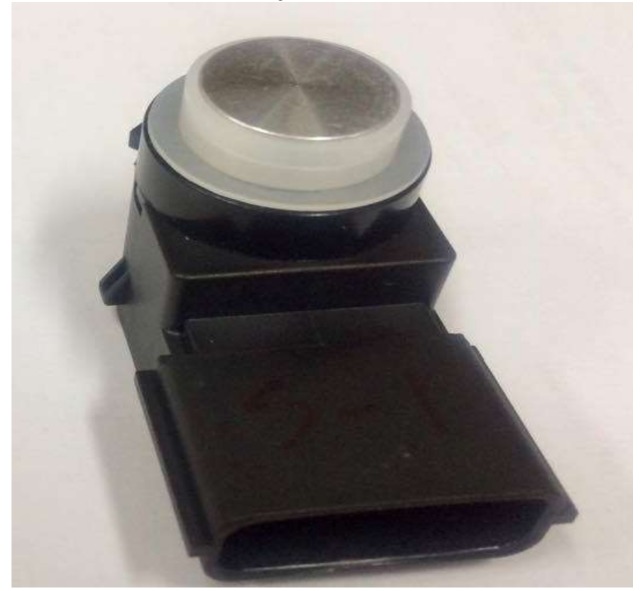
An actual ultrasonic sensor for in-air applications.

**Figure 12:**
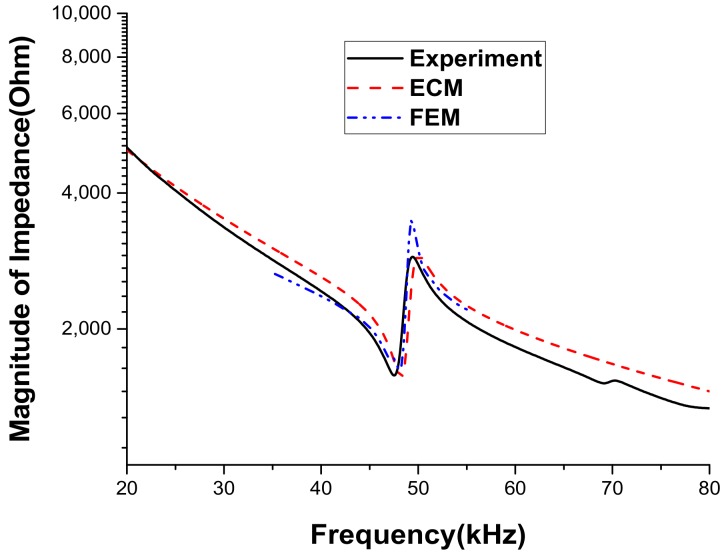
Comparison of the impedance spectra of the ultrasonic sensor obtained by different methods.

The validity of the ECA was verified by comparing the analysis results with those from the FEA in the previous section. In order to validate the ECA results more realistically, the impedance spectrum and beam pattern of an actual ultrasonic sensor in air were measured experimentally and compared with those from the ECA. Figure 11 is a photograph of a typical in-air ultrasonic sensor for automobiles. This ultrasonic sensor has exactly the same multilayered structure as that in Figure 2. The dimensions and materials of the sensor are identical to those in Table 1 and Table 2. Hence, the equivalent circuit parameters derived in Section 3 can be used to represent the properties of this ultrasonic sensor. However, in order to control the magnitude of the impedance at the resonance and anti-resonance frequencies of the structure, two resistors *R*_0_ and *R*_A_ were added to the electrical and acoustical branches of the equivalent circuit in Figure 3, respectively. The electrical resistor *R*_0_ was assumed to be 2 kΩ whereas the acoustic resistor *R*_A_ was calculated using the relationship with a damping ratio (*ζ*), given as *R*_A_ = 2*ζ* (*M_A_/C_A_*)^1/2^ [37]. The term *ζ* that is typically an empirical value determined via experiments was assumed to be 0.017 [37]. The impedance spectrum of the ultrasonic sensor was measured using the impedance analyzer Agilent 4294A (Agilent Technologies, CA, USA). Figure 12 compares the measured impedance spectrum with those calculated using the ECM and FEM. The resonance frequency from the FEA is 48.0 kHz whereas that from the ECM is 48.2 kHz and the difference is only 0.42%. The resonance frequency from the measurement is 47.5 kHz, which differs from that from the ECA by merely 1.4%. The discrepancy is considered to be due to the tolerance in fabricating the ultrasonic sensor. This agreement between the results from the ECA, the FEA, and the measurement confirms the validity of the ECM.

**Figure 13:**
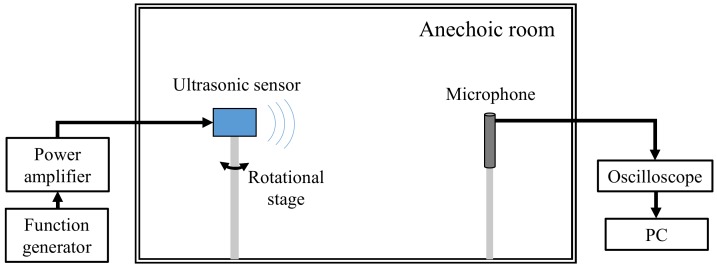
Schematic of the experimental setup to measure the beam pattern of the ultrasonic sensor.

**Figure 14:**
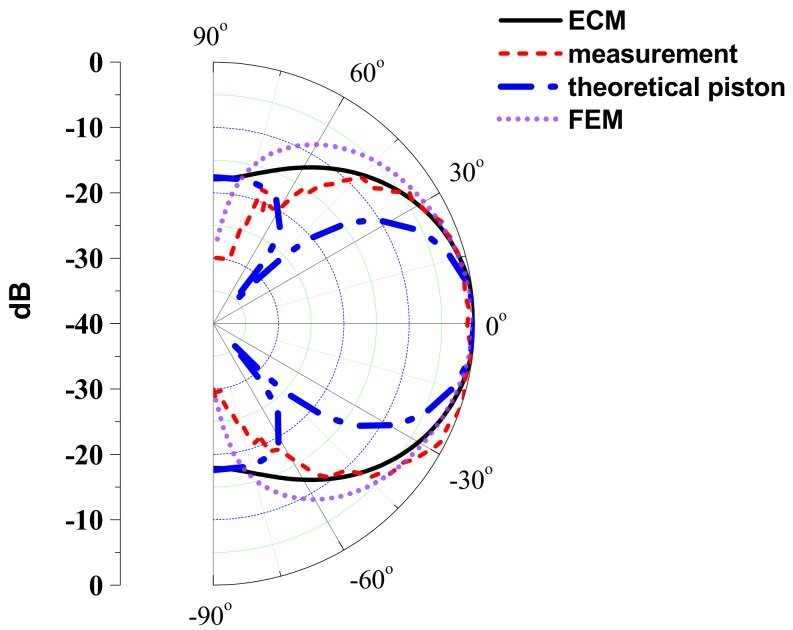
Comparison between analytical, simulated, and measured beam patterns of the ultrasonic sensor.

The beam pattern of the ultrasonic sensor was also measured and compared with the analytical beam pattern derived in Section 4. Schematic of the experimental setup to measure the beam pattern is shown in Figure 13. The ultrasonic sensor was placed on a rotational platform in an anechoic chamber and an electric voltage pulse was applied to excite the sensor. The radiated sound pressure was measured with a microphone located at a far-field distance from the ultrasonic sensor. The measured beam pattern is compared with the analytical beam pattern computed using Equation (43) and that using the FEM as shown in Figure 14. Figure 14 also compares the beam pattern obtained by considering the vibrational plate as a theoretical circular piston source of the same dimension. Comparison with the piston source is conducted because, in many practical cases, the beam pattern of a small ultrasonic sensor is approximated using the theoretical equation for the piston source [36]. It is clear from Figure 14 that the beam pattern obtained by ECM is in close agreement with those obtained by the FEM and the measurement. The beam widths of the ultrasonic sensor calculated using the ECM and the FEM and that measured experimentally are 58.2°, 62.8° and 61°, respectively. The difference between the measured and the two analyzed beam widths is less than 4.6%, which is attributed to the experimental tolerance in fabricating the actuator ultrasonic sensor and evaluating the beam pattern. On the other hand, the beam pattern of the circular piston source is significantly different from the measured pattern. This result confirms the accuracy and efficacy of the ECM developed in this study in estimating the performance of the actual ultrasonic sensor.

## 7. Conclusions

We developed the ECM to investigate the electroacoustic behavior of the piezoelectric multilayered structure as an ultrasonic sensor. The vertical deflection of the multilayered structure was derived as a function of loaded pressure and voltage using the equilibrium equations of the axisymmetric structure. The novelty of this work lies in that the vertical deflection includes the effect of piezoelectric forces and moments, which was followed by the evaluation of equivalent circuit parameters. Acoustic characteristics of the piezoelectric multilayered structure were then analyzed using the equivalent circuit parameters and the effects of geometrical variations of the individual components on the performance of the ultrasonic sensor were studied. The analyzed characteristics of the ultrasonic sensor included resonance frequency and acoustical beam pattern. The validity and efficacy of the ECM were verified by comparing the results with those from the FEA and an experimental characterization of an actual ultrasonic sensor. The ECM presented in this study can estimate the characteristics of a piezoelectric multilayered structure more accurately in comparison with the precedent theoretical method of the authors [25].

## Figures and Tables

**Table 1:** Material properties of the multilayered structure [25].

Component	Density (kg/m^3^)	Young’s Modulus (GPa)	Poisson’s Ratio	Piezoelectric Constant *d*_31_ (pm/V)
Metallic plate (Al)	2690	70	0.34	
Bonding layer	1175	4	0.34	
Piezoceramic (PZT-5A)	7750	61	0.34	−171
Backing layer	25.6	0.0025	0.34	

**Table 2:** Initial dimension of the multilayered structure (unit: mm) [25].

Radius of Vibrational Plate	Radius of Piezoceramic	Thickness of Vibrational Plate	Thickness of Piezoceramic	Thickness of Backing Layer	Thickness of Bonding Layer
6.14	4.5	0.72	0.24	10.54	0.005
